# Reply to: Ocean afforestation is a potentially effective way to remove carbon dioxide

**DOI:** 10.1038/s41467-023-39927-y

**Published:** 2023-07-20

**Authors:** Lennart T. Bach, Veronica Tamsitt, Jim Gower, Catriona L. Hurd, John A. Raven, Philip W. Boyd

**Affiliations:** 1 Institute for Marine and Antarctic Studies, University of Tasmania, Hobart, TAS Australia; 2 College of Marine Science, University of South Florida, St Petersberg, FL USA; 3 Fisheries and Oceans Canada, North Saanich, BC Canada; 4 Division of Plant Sciences, University of Dundee at the James Hutton Institute, Invergowrie, Dundee, UK; 5 Climate Change Cluster, University of Technology, Ultimo, Sydney, Australia; 6 School of Biological Sciences, University of Western Australia, Crawley, WA Australia

**Keywords:** Carbon cycle, Ocean sciences

**replying to** W.-L. Wang et al. *Nature Communications* 10.1038/s41467-023-39926-z (2023)

We thank Wang et al. for critically assessing our study^1^. Their main critique concerns our estimates of the calcification discount to the ocean afforestation CDR potential and our omission of other carbon-fixing processes provided by *Sargassum*. We will respond consecutively and systematically refute the claims made by Wang et al.^1^.

## Calcification

Wang et al. suggest that our estimated maximum discount to the CDR potential via the calcification feedback is based on an outlier^1^. The value we used (21.4%) is the upper bound for the CaCO_3_ wet weight biomass percentage^2^ for a series of 13 Sargasso Sea samples ranging from 4.3–21.4%^3^. We used the Grubbs test which did not identify 21.4% as an outlier. Wang et al.^1^ point us towards a valuable reference that reported 23 values with lower CaCO_3_ wet weight biomass percentage (0.94–3%) in *Sargassum* collected at the Mexican coast^4^. However, this reference was not published when our analysis was done. Including their values^4^ extends the range towards the low end, i.e., towards the lower-bound scenario for ocean afforestation. The updated range based on these two published studies is a 1.3–57% discount (it was 7–57% in Bach et al.^2^). The conclusion drawn from this range is fully consistent with the concluding remark in our calcification section where we stated: “The calcification offset could range from being negligible to being a major factor reducing the CDR efficiency of ocean afforestation”^2^. We emphasise that biofouling by bryozoans and other calcifiers is a widespread and well-documented problem in seaweed aquaculture^5–7^, so that the calcification discount to ocean afforestation should not be readily dismissed.

Wang et al.^1^ also suggest that our referenced value (0.01) for the phytoplankton PIC:POC ratio (mol:mol) is too low, and suggest 0.01–0.07 based on references used by us^8^, and 0.23–0.24 based on another reference^9^. We did not use these high values^9^ because Sarmiento et al. provided good evidence for their conclusion that: “the [PIC:POC] export ratio is unlikely to be anywhere near as large as the value of 0.20–0.25 used in some modelling studies”^8^ (Sarmiento et al.’s global average was 0.06)^8^. The 0.01 was chosen as this value is largely consistent for subtropical North Atlantic based upon both direct measurements^10^ and PIC:POC export ratio estimates^8^. When we repeat our best-case calculation here with the lower bound for CaCO_3_ wet weight biomass percentage (0.94%) and an upper bound phytoplankton PIC:POC of 0.07 suggested by Wang et al.^1^, the CDR efficacy of ocean afforestation would be reduced by 10–100%, as opposed to 20–100% reported in Bach et al.^2^.

## Nutrient reallocation

**Fig. 1:**
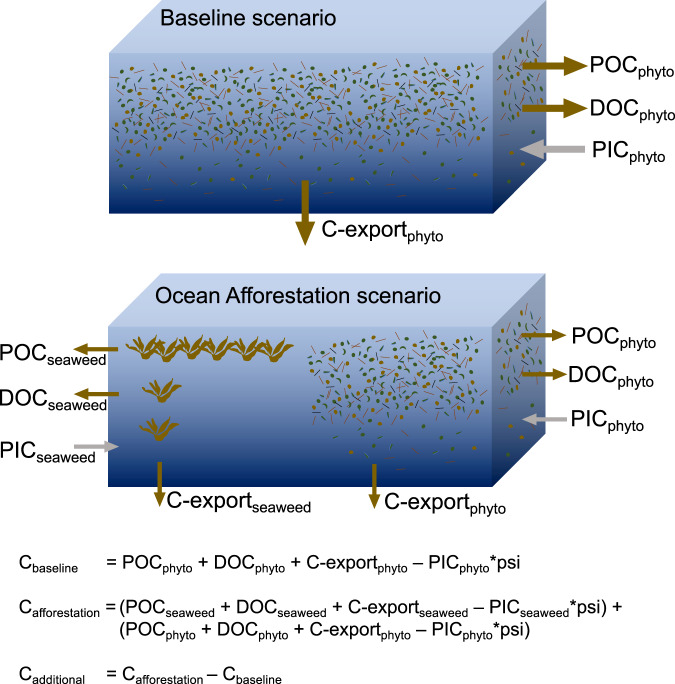
Framework to assess the additional carbon sequestration (C_additional_) with ocean afforestation. The framework considers predominant carbon pools and sequestration pathways of a baseline ecosystem driven by phytoplankton (upper illustration). Carbon pools and sequestration pathways are for example particulate organic carbon (POC), dissolved organic carbon (DOC), carbon export (all three are CO_2_ sinks) and particulate inorganic carbon (PIC, a CO_2_ source by reducing seawater alkalinity by a factor psi^18^). For ocean afforestation (lower illustration), a proportion of the nutrient inventory, which controls the magnitude of all carbon pools and fluxes, are diverted to the corresponding seaweed carbon pools and fluxes. This reduction of a former C-sink has to be considered for the calculation of how much ocean afforestation can add to the marine biotic carbon sink as shown in equations at the bottom. These equations are an extended version of Eq. 1 in Bach et al.^2^ to account for comments by Wang et al.^1^. The equations include simplifications, for example that different pools/fluxes sequester carbon for different timescales. Also, it assumes linearity between the baseline and the ocean afforestation scenario although the implementation of ocean afforestation may have synergistic/antagonistic effects on phytoplankton carbon sequestration. Consideration of these and other variables will further complicate the calculation of additionality.

Wang et al.^1^ argue about the lower-bound calcification discount, which has a limited influence on the overall conclusion of this study, even when their estimates are considered (see previous section). However, they misjudge the fundamental constraint of ocean afforestation - nutrient reallocation from phytoplankton to macroalgae. Although they address nutrient reallocation specifically, they contextualise it in the marginal role this mechanism has in reduction of phytoplankton calcification (see Fig. 2C in Bach et al.^2^). However, the most fundamental biogeochemical constraint of ocean afforestation is that it is fuelled by nutrients, which already fuel an existing carbon sink, the biological carbon pump driven by phytoplankton. In the (sub)tropical North Atlantic, these nutrients are largely depleted, meaning the biological carbon pump already operates at almost maximum efficiency^11,12^. Introducing seaweeds to this region means that part of the nutrient inventory utilised by phytoplankton may then be utilised by seaweeds^13^. Thus, by adding a new biotic carbon sink (seaweeds), an existing one is reduced (phytoplankton) (Fig. 1).

Thus, for ocean afforestation to work, it must sequester more carbon than the baseline ecosystem (phytoplankton) already does currently. Since phytoplankton keeps the subtropical surface ocean relatively “clean” of nutrients (i.e. the biological carbon pump operates at maximum efficiency^11,12^), seaweeds would have to sequester more carbon with the available amount of limiting nutrients. This is generally the case for *Sargassum* reflected in a 2 to 13.5-fold higher C:N (0.7 to 8-fold for C:P)^14^, but the baseline (phytoplankton C sequestration) must be subtracted from seaweed C sequestration to estimate the added value (i.e., additionality) of the seaweed carbon sink (Fig. 1). Wang et al. report other carbon sequestration mechanisms such as the release of dissolved organic carbon (DOC) and suspended particles (sPOC), which could add to the ocean afforestation potential^1^. However, the amount of DOC and sPOC formed is not such a relevant metric. Instead, Wang et al., should consider: Is the release of DOC by seaweeds sequestering more carbon than the release of DOC by the phytoplankton that existed before the nutrients were re-allocated to fuel seaweed growth? And: Is the production of sPOC sequestering more carbon than the continuous aggregation and export of organic matter by phytoplankton that existed before the nutrients were reallocated to fuel seaweed growth? Assessing ocean afforestation through the lens of additionality illustrates the complexity of the nutrient reallocation problem when it is applied to the many pathways within the ocean carbon cycle (termed monitoring, reporting, and verification (MRV)). To demonstrate that ocean afforestation works, we not only have to measure carbon sequestration by seaweeds but first must assess the baseline carbon sequestration that existed before phytoplankton was partially replaced by seaweeds (Fig. 1). We have put forward this argument on MRV within the DOC section of the original paper^2^. It is possible that this important point may have been overlooked, but we have clarified it here.

Quite en passant, Wang et al. suggest that ocean afforestation will not work without nutrient fertilisation, provided externally or via artificial upwelling^1^. This statement is remarkable because Wang et al. swiftly convert ocean afforestation into a sub-category of ocean nutrient fertilisation in which carbon sequestration is enabled by the supply of additional nutrients and then supposed to be boosted by seaweeds. Their assertion adds another layer of complexity to the assessment of ‘ocean fertilisation with afforestation’ because not only is ocean biology manipulated but also ocean chemistry. The recycling of nutrients from seaweed biomass they mention would indeed alleviate the CDR discount via nutrient reallocation, as we have already emphasised in our paper^2^. However, Wang et al. repeat this hypothetical solution without any indication whether such nutrient-extracting/carbon-purifying factories are feasible. Providing nutrients via artificial upwelling to enhance seaweed growth would increase complexity further due to the additional manipulation of ocean physics. Artificial upwelling has been shown to induce major side-effects^15^. Furthermore, nutrients upwelled to fuel seaweed growth would no longer be available to phytoplankton assemblages downstream at the time they are naturally upwelled. Thus, as argued in our paper^2^, fuelling ocean afforestation by artificial upwelling just shifts the problem of nutrient reallocation in space and time.

## Air-sea CO_2_ equilibration timescales

Wang et al. argue that our calculations of air-sea equilibration timescales using the methodology (and some data) from Jones et al.^16^, are inappropriate. Their argument is that mixed layer depth may be different when floating seaweeds are present but do not provide any evidence to support this claim. Instead, they conclude that a carbon cycle model should be used for this assessment. But again, they provide no evidence for why such a model should provide more reliable timescales than the approach by Jones et al.^16^, which is based on climatological data and offline modelling with virtual particle tracking. Ultimately, we note that even if the timescales were somewhat shorter than 2.5–18 months (due to a shallower mixed-layer), the problem discussed in our paper would remain unless frameworks are developed and adopted which could solve it^17^.

## Conclusion

Wang et al., provided some recently published data on seaweed calcification rates, that when included in a re-analysis did not alter the conclusions drawn by Bach et al.^2^. Current evidence shows that the CDR potential of ocean afforestation is much less than its advocates suggest, due to fundamental biogeochemical constraints. We stand by our original conclusion^2^ and still consider other (non-biotic) CDR methods with less biogeochemical complexity, to have a higher potential for delivering clearly verifiable CDR in the near-term.

## Data Availability

The manuscript contains no new data or code.
